# The Necessity for the Human Element

**Published:** 1919-11-22

**Authors:** 


### WORK AND WELFARE.

### The Necessity for the Human Element.

Never before in the history of this country have social conditions reached so acute and serious a phase as at the present time; the hour is very critical, and the whole position of labour is undergoing a momentous transition. In every branch of industry there is an increasing tendency to amalgamate the unions into one great incorporate whole, thereby dividing the community into two great camps, the one a labour, the other a so-called non-labour camp. The word "labour" has, like so many other words in our language, come to lose its original signification. The expression "Labour Party" suggests the industrial or working-class section of the community, as against the remainder, who, presumably, do not work for their living. That this explanation is grossly misleading must be obvious to all thinking men and women, for it is only a very small minority who do not, in some way or another, use their mental and physical powers for the purposes of earning a livelihood.

### Capital and Labour.

We believe that it is essential for the co-operation of capital and labour, or rather, shall we say, employer and employed, that this misunderstanding should be once and for all rectified. Now to rectify a well-established opinion it is necessary, first of all, carefully to examine the whole position, both in principle and detail, and from both sides, and to do this with an open mind, so far as such impartiality is possible with human intelligence. Secondly, that having examined and found conditions which must militate against true reciprocity between employer and employed it is then our bounden duty to apply at once the necessary remedy, if it can be discovered. Social disease requires just as careful diagnosis and treatment as is the case with pathological processes in the human body. Too often have remedies been applied which have only relieved symptoms without curing the disease, for, sooner or later, these symptoms recur, with often increased virulence.

It is a well-established fact that the best work can only be produced under the most favourable conditions. Man is not alone in this respect, for the same rule applies to all the members of the animal and vegetable kingdoms. A plant deprived of oxygen will die; a fish removed from its natural habitat rapidly ceases to exist; and the human being bereft of light and sunshine, and of the necessary amount of protein, carbohydrate, and fat in his diet will, sooner or later, cease to become an efficient citizen.

Again, in order to teach the average human being the inestimable value of cleanliness it is not sufficient merely to point out to him its many advantages; we must provide the means for being cleanly. For some years painters have learnt the lesson, and the use of a nail-brush and soap has undoubtedly saved many of this class of worker from lead toxæmia.

The value of proper nutritious food cannot be too often taught and demonstrated, for the daily meals of the average girl worker have, in innumerable cases, proved but a travesty of a repast, with the inevitable result.

Another point that demands careful investigation is the maximum amount of work that should be demanded from any one worker; this is a most difficult question to decide, for no two persons possess the same capabilities for mental or physical labour; hence there cannot be a definite and absolute standard. Again, man differs greatly in his practical adaptation to his work; one will perform in one hour as much as three or four others could in the same time turn out by their united efforts; for instance, one understands method, another does not, and so on.

### In The Workshop.

In a workshop the number of working hours should be determined by the average. There is many a man who is capable of performing more than eight hours of good work per day, as doctors and others know well, and thus it follows that arbitrary limitation prevents the greatest possible production.

Strange as it may seem to many of our citizens, there is, undoubtedly, a certain amount of danger to the individual in his carrying out too small an amount of work; there is nothing more disastrous to the average human being than idleness—it breeds discontent, and discontent leads to crime. It requires a very clever brain to withstand the degenerating influence of idleness. There are in every city to be found a certain number of loafers to whom the very thought of honest work is a nightmare, and whose chief pleasure consists in reaping the benefits of others' labour, only too often by most disreputable means.

The application of practical remedies has already been partially discussed, and a few words are still necessary concerning feeding. Now it is both foolish and cruel to advise a man to feed properly when he does not possess the adequate means of procuring that sustenance, and therefore it is the bounden duty of the State to ensure that every worker receives a wage commensurate with his actual needs; that is to say, not less than a living minimum wage.

Finally, the most practical treatment of this disease called industrial unrest is to be found in the real co-operation between all classes; in a state of true democracy there should be neither autocracy on the one hand nor greed on the other. There can never be a maximum output of labour until there is a bond of fellowship and recognition that no work is degrading provided that it is honest, and so for the benefit of the nation. Every one of us is an offspring of the country that gave us birth, and it should be the greatest ideal of all of us to render to our beloved parent what is best and noblest in us. Thus, and thus only, will this mighty nation continue to hold its proud position in the world.

